# Re-evaluating the importance of carbohydrates as regenerative biomaterials

**DOI:** 10.1093/rb/rby023

**Published:** 2018-11-14

**Authors:** Heidi F Oldenkamp, Julia E Vela Ramirez, Nicholas A Peppas

**Affiliations:** 1 Institute for Biomaterials, Drug Delivery, and Regenerative Medicine, The University of Texas at Austin, Austin, TX, USA; 2 McKetta Department of Chemical Engineering, The University of Texas at Austin, Austin, TX, USA; 3 Department of Biomedical Engineering, The University of Texas at Austin, Austin, TX, USA; 4 Department of Pediatrics, Dell Medical School, The University of Texas at Austin, Austin, TX, USA; 5 Department of Surgery and Perioperative Care, Dell Medical School, The University of Texas at Austin, Austin, TX, USA; 6 Division of Molecular Pharmaceutics and Drug Delivery, College of Pharmacy, The University of Texas at Austin, Austin, TX, USA

**Keywords:** carbohydrate, regenerative biomaterials, polysaccharide, tissue engineering

## Introduction

Carbohydrates are the most abundant natural biomaterials in the world. By interacting with cells of a variety of levels, they take part in essential functions of organisms including cellular communication, inflammation, infection development and disease. These carbohydrate–cell interactions occur on a variety of levels through glycoconjugates such as glycolipids, glycosaminoglycans (GAGs), glycoproteins and proteoglycans [1–4]. The roles of carbohydrates in biological systems pose them as some of the most sought-after biomaterials. The use of these multifaceted molecules provides the opportunity to tailor desired responses depending on the target application [1–5].

Genomics and proteomics have been oriented to discover the information contained in either the genetic code of different organisms or in the amino acid sequences of proteins, respectively. However, the area of glycomics is still very new, with its goal to achieve the level of understanding with glycans, as with other natural molecules [4, 6, 7]. In particular, glycomics researchers hypothesize that the capabilities of polysaccharides to store biological information is more extensive than other molecules, due to the varied glycosidic bonds and interactions between saccharides, as well as the different conformational structures that they can adopt [1, 3, 4, 7]. Elucidation of the complex interactions between specific polysaccharide sequences and other biomolecules is one of the main goals of glycomics [2]. This information would be extremely valuable for the design of novel solutions to biological and chemical challenges.

Advances in glycomics research would be of great advantage to the biochemical, medical and materials science fields. Understanding the functions of polysaccharides in cell–cell interactions, inflammation signaling and the progression of different diseases will enable the rational-design of biomaterial platforms, targeting molecules or treatments [8]. Moreover, the potential applications of this knowledge in drug delivery and tissue engineering are innumerable. Therefore, this perspective will discuss some of the current applications of polysaccharides in these fields and discuss new frontiers in polysaccharide biomaterials research.

## Carbohydrate and polysaccharides as biomaterials

Carbohydrates interact with other carbohydrates and proteins thus playing a role in cellular adhesion and differentiation [9, 10]. Specifically, carbohydrate–carbohydrate interactions have been shown to impact cellular recognition and adhesion [9–13]. Likewise, carbohydrate–protein interactions have been studied extensively and have demonstrated to influence infection, inflammation and cellular adhesion [3, 14, 15]. Even though carbohydrate interactions are typically weak [1, 11, 16, 17], multivalent interactions, where multiple polysaccharides are involved, enhance the interaction strength [10, 18–20]. Both ligand-induced protein clustering and the chelate effect, a phenomenon in which an initial interaction is followed by multiple interactions, combine to strengthen multivalent polysaccharide interactions [1, 18, 21]. Carbohydrates are versatile biomaterials due to the effects of their interactions with other polysaccharides and other biomolecules.

Polysaccharides, such as chitosan, hyaluronic acid and alginates, have been used as biomaterials for many years. Some of their advantages are their capability to support cell growth, advantageous mechanical properties, ability to form hydrogels and biocompatibility [22–26]. Drug delivery and tissue engineering studies have utilized these biomaterials for specific applications. In particular, drug delivery has explored polysaccharide polymers as carriers, as targeting agents and as targeted ligands. Tissue engineering has used polysaccharide biomaterials to mimic the cellular extracellular matrix and serve as scaffolds for controlled tissue growth. In addition, the use of polysaccharides in scaffold modification for their customization as cellular matrices has been explored in tissue engineering. Here, we expand on these applications following a brief description of chitosan, hyaluronic acid and alginate.

### Chitosan

**Figure 1:**
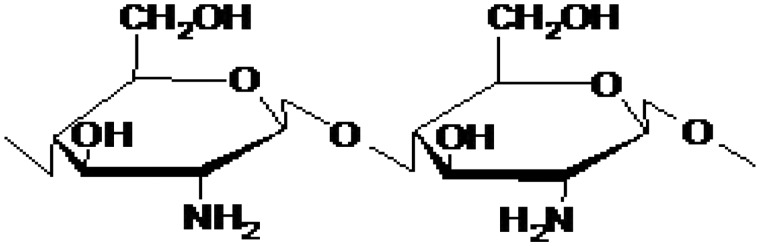
Chitosan structure

Chitosan, a linear polysaccharide consisting of β(1–4) linked d-glucosamine residues and *N*-acetyl-glucosamine residues, is a de-acetylated chitin derivative [26] (Fig. 1). It is semi-crystalline in form and may be formed into hydrogels at pH higher than 6.5. The molecular weight of chitosan may range from 50 to 1000 kDa and it is positively charged in its native form. The primary amine groups allow for easy modification, such as conjugation with sulfate groups, which causes that the molecule becomes negatively charged. It is biodegradable, biocompatible and can be water-soluble or insoluble depending on its form [23, 25–27].

### Hyaluronic acid

**Figure 2:**
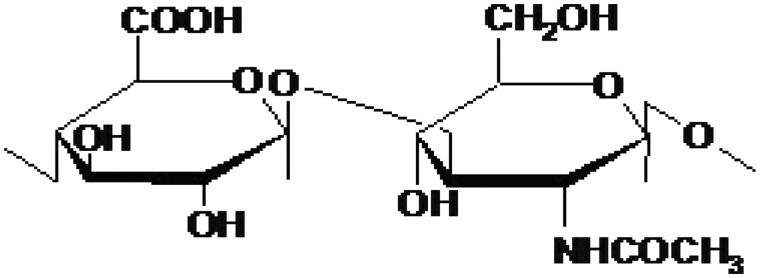
The molecular structure of hyaluronic acid

Hyaluronic acid is a GAG that is naturally found in extracellular matrices. Unlike chitosan, hyaluronic acid is present in humans and it is immunoneutral. It is composed of alternating units of d-glucuronic acid and *N*-acetyl-d-glucosamine as shown in Fig. 2. Hyaluronic acid is a highly hydrated, negatively charged polymer with a high molecular weight over 1000 kDa. Like chitosan, hyaluronic acid can be modified to yield desired properties, such as changing the hydrophobicity or biological activity. Hyaluronic acid has been shown to be biocompatible, biodegradable and have wound healing properties [24, 28, 29].

### Alginates

**Figure 3:**
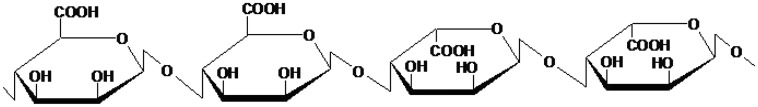
The structure of alginate

Alginates are derived from brown algae and are composed of (1–4)-linked β-d-mannuronic acid and α-l-guluronic acid residues (Fig. 3). These two groups can be arranged consecutively or alternating. The composition and arrangement of these residues leads to varying physical and mechanical properties. The molecular weight ranges from 32 to 400 kDa that is much smaller than hyaluronic acid [22, 30]. Alginates can be ionically or covalently cross-linked [31]. Alginates are also biodegradable and biocompatible under certain conditions, but its composition can affect these properties [30, 32].

It should be noted that not all polysaccharides are suitable to be used as biomaterials. For instance, carrageenan, a polysaccharide derived from red seaweed and widely used as a food additive, has shown to lead to adverse effects as a biomaterial [33]. Hence, all new biomaterials should be comprehensively tested to evaluate both *in vitro* and *in vivo* biocompatibility [34]. However, when inflammation or antigenic cues are beneficial, glycans or glycoproteins that elicit immune responses have been incorporated into different systems [35–37].

## Polysaccharides as carriers for drug delivery: Back to the roots

**Figure 4:**
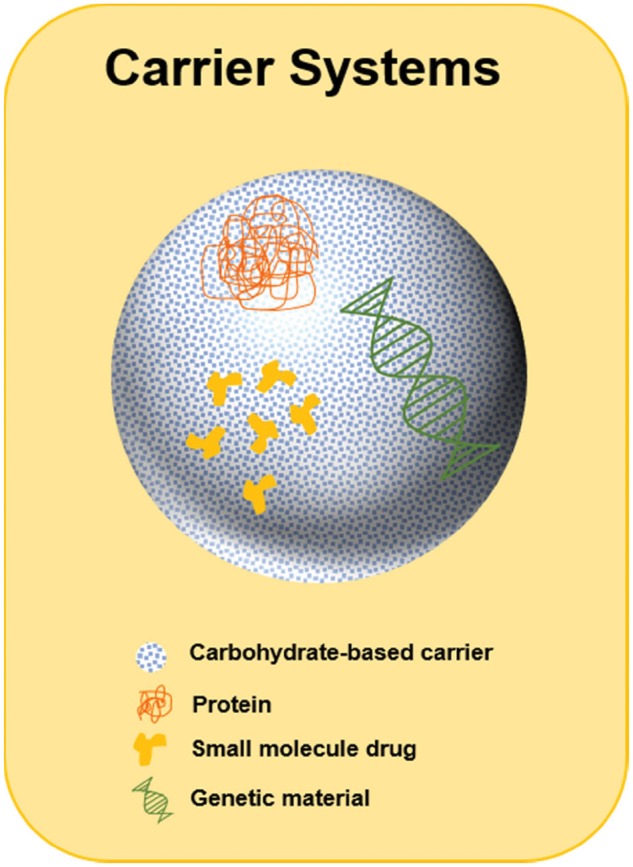
Carbohydrate-based carrier systems. Carbohydrate-based biomaterials can be used as carriers for drugs, such as proteins, small molecules or genetic material

These natural polysaccharide polymers have been used in drug delivery because of their known biocompatibilities and biodegradation. Applications in drug delivery include using these polymers as drug carriers (Fig. 4) and using these and other carbohydrates for the targeted delivery of drugs to specific locations.

### Polysaccharides as carrier systems

Positively charged chitosan-based drug delivery platforms have been successfully developed, particularly for use as gene-delivery systems due to their ability to complex with negatively charged DNA [38, 39]. In one study, Roy *et al.* delivered gene therapy-based vaccines against peanut allergy using chitosan nanoparticles [40]. A genetically engineered vaccine was prepared by complexing plasmid DNA with chitosan. The resulting nanoparticles were then orally administered to mice in a sensitization study. After an intraperitoneal challenge with Arah2 protein, the anaphylactic response of the vaccinated mice was significantly lower than both the non-immunized and the vaccine-only control groups [40]. These results showed the potential of DNA vaccination using chitosan-based oral drug delivery systems.

In another study, Senel *et al.* [41] evaluated the potential of a buccal drug delivery platform for proteins utilizing chitosan. In this work, chitosan gels demonstrated their ability to increase the permeability of porcine oral mucosa to transforming growth factor-β with an *in vitro* model [41]. Besides this application, Janes and Alonso [42] described the myriad of other drugs that have been delivered using chitosan-based carriers, such as the proteins bovine serum albumin and insulin [42].

Hyaluronic acid has been widely explored for the delivery of anticancer agents due to its ability to target the CD44 receptor, which is associated with cancer. Using hyaluronic acid as a carrier for hydrophobic drugs increases their solubility and may increase the circulation time of many drugs, similarly to polyethylene glycol [43].

Alginates have also been used in the development of platforms for drug delivery [44, 45]. In the studies by Bouhadir *et al.* a new monomer was synthesized by the oxidation of sodium alginate [46]. The resulting modified polysaccharide was then engineered for the delivery of antineoplastic agents. The three drugs were each released via three distinct mechanisms: diffusion, ionic dissociation and covalent bond degradation. Proteins such as fibroblast growth factor, bovine serum albumin, nerve growth factor and interleukin-2 have been successfully delivered using alginate-based carriers [30, 47].

Besides the aforementioned materials, there are other natural polysaccharides that have been explored as drug delivery systems, including amylose, dextran and pectin. These have been further discussed in the works by Sinha and Kumria [48] and Vandamme *et al.* [49].

### Carbohydrates may function better as carriers for targeted delivery

**Figure 5:**
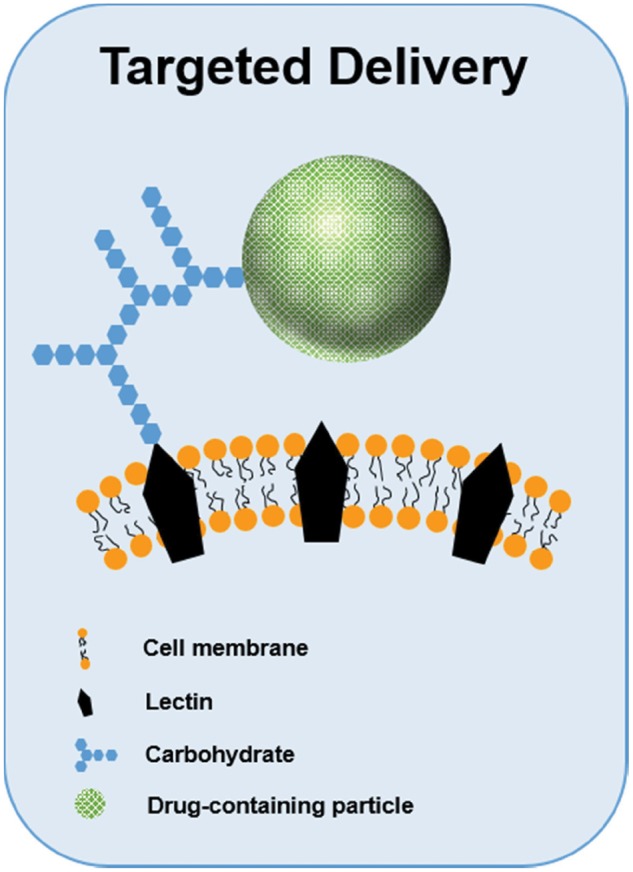
Lectin–carbohydrate interactions are commonly exploited for targeted drug delivery. Drug-loaded particles decorated with carbohydrates can be used to target specific cells via these interactions

Targeted drug delivery systems commonly utilize lectin–carbohydrate interactions. As previously described, the strength and specificity of these interactions can change depending on multivalency. This is of particular importance in the design of drug delivery systems since carbohydrates can be used to target lectin receptors in selected cells and tissues and improve uptake [50]. The innovative research into polysaccharide targeting systems shows incredible potential to revolutionize the drug delivery field [51]. The use of lectins to target specific tissues has been included in the development of different drug delivery systems as shown in Fig. 5 [50, 52, 53]. For example, the work of Umamaheshwari *et al.* showed that gliadin nanoparticles decorated with lectins could be used to avoid the binding of *H. pylori* strains to human stomach cells [54]. Lectins have also been used to overcome the challenges presented by the intestinal mucosa by targeting epithelial M-cells and Caco-2 cells [55–59]. Wood *et al.* delivered insulin to carbohydrate residues found in the intestinal mucosa using a wheat germ agglutinin-functionalized complexation hydrogel drug delivery system [60, 61]. Results showed increased insulin transport across the intestinal epithelium and indicated that wheat germ agglutinin (a lectin) improved the bioavailability of insulin [62].

Xu *et al.* investigated the use of wheat germ agglutinin, tomato lectin and Ulex europaeus agglutinin 1 (UEA1) to enhance the delivery of insulin to the bloodstream [63]. Lectin-modified liposomes were loaded with insulin, and *in vivo* delivery studies demonstrated that while all three lectins increased insulin delivery compared with controls, wheat germ agglutinin increased it the most [63].

As shown by Hama *et al.*, lectins can also be used to deliver imaging modalities to cancerous cells [64, 65]. In this study, fluorescently labeled avidin was used to bind asialo-receptors (surface lectins) on cancer cell lines. Results showed that this technique, along with fluorescence imaging, could be used to detect cancerous cells. Images from nine different cell lines were observed, including ovarian, colon and breast cancers, with all results indicating that this method could be used to deliver imaging modalities to cancerous cells in a targeted manner.

Targeted drug delivery systems that use carbohydrate moieties have also been studied [35, 66–68]. Cho *et al.* used galactose-containing nanoparticles to target lectin-based receptors on hepatocytes [67]. The poly(l-lactic acid) particles used in this study were coated with polystyrene and galactose and loaded with *trans*-retinoic acid [67]. Results showed that these particles were successful in targeting hepatic cells for the delivery of this drug. In another study, the delivery of anti-inflammatory drugs to areas of chronic inflammation using lectin–ligand chemistry was evaluated [69]. Eniola *et al.* used carbohydrates to target P-selectin, a lectin expressed by endothelial cells that plays an important part in blood vessel inflammation by supporting the rolling of neutrophils [69]. Neutrophil rolling is mediated by Sialyl–Lewis_x_ (sLe_x_), a sialylated and fucosylated carbohydrate. Eniola *et al.* worked to deliver anti-inflammatory drugs by mimicking this neutrophil rolling by synthesizing sLe_x_-decorated PLGA microparticles [69]. They conducted laminar flow tests using this drug delivery system and P-selectin-coated surfaces [69]. Results showed that the density of P-selectin molecules conjugated to the surface of the microspheres dictated the degree of neutrophil rolling on the coated surfaces [69]. By demonstrating that lectins on cell surfaces can be targeted by drug delivery systems containing carbohydrates, these studies highlight the promise of utilizing this interaction between lectins and carbohydrates for other types of cell-targeted delivery.

### Alternative carbohydrate-based targeting strategies

Beyond the use of carbohydrates as targeting agents or delivery vehicles, exploration of alternative applications for these molecules in drug delivery has been performed. For instance, Mahal *et al.* described an innovative approach to improve the selectivity of drug carriers by selectively decorating the surfaces of cells with polysaccharides [70, 71]. They converted ketone-containing *N*-acetylmannosamines into sialic acids and utilized natural oligosaccharide metabolism to obtain ketone groups on the cell surface [70]. These ketone groups were then biotinylated, which enabled the targeted killing of the cells with toxin–avidin conjugates [70]. The results of this study suggest that this strategy could also be used to target and kill cells that express sialic acid at a high level, such as tumor cells [70].

Furthermore, in the development of vaccine delivery vehicles, the use of carbohydrates as pathogen-mimicking moieties to elicit robust and safe immune responses has been explored [72–74]. This is achieved either by the carbohydrate-functionalization of the antigen and/or the delivery platform and with the use of sugars as antigens [75, 76]. The findings of these studies have demonstrated the abilities of α-1,2-linked dimannosides and β-galactosides to stimulate immune cells and to modify the protein adsorption patterns onto nano-adjuvants, which are important in the development of immunity [72, 77]. These efforts have been aimed at mimicking glycoproteins in viral or bacterial diseases (i.e. HIV) [78–83], and tumor-associated carbohydrate antigens [84, 85]. These studies suggested that the use of oligosaccharides for the enhancement of the adjuvanticity of drug delivery vehicles is an exciting research area that can have successful outcomes against a variety of diseases.

## Polysaccharide biomaterials as scaffolds in tissue engineering

Polysaccharide biomaterials have been applied to the tissue engineering field in various ways. Since scaffold engineering is almost synonymous with tissue engineering, the selection of the biomaterials for scaffold design plays an integral role in the overall success of the implant [86–91]. The physicochemical characteristics of the material modulate cellular adhesion, cellular proliferation, and the inflammatory response toward the scaffold. Biodegradable materials are especially attractive for tissue engineering scaffolds because they may help to limit a chronic foreign-body response that could ensue from a permanent implant [92]. Polysaccharide biomaterials have, therefore, been used in a variety of tissue engineering fields including skin, cartilage, cardiovascular, neural and hepatics.

### Skin regeneration and wound healing applications

The importance of the function of the skin as an environmental barrier is demonstrated by serious illness following the loss of integrity of large portions of skin. Patients with skin damage due to burns and other deep injuries to require grafts if the loss of full-thickness skin exceeds 4 cm in diameter, while smaller injuries likely only require wound dressings [93]. The development of skin replacements for these cases has proved difficult, partially due to appendages that project through its multiple layers [94]. Tissue engineered skin substitutes are currently one of the most commonly explored strategies for wound healing and skin regeneration. In fact, the first tissue-engineered products available to patients were skin replacements [93]. The most important properties of these materials include their ability to provide environmental protection while allowing water permeation, pain reduction and healing promotion.

**Figure 6:**
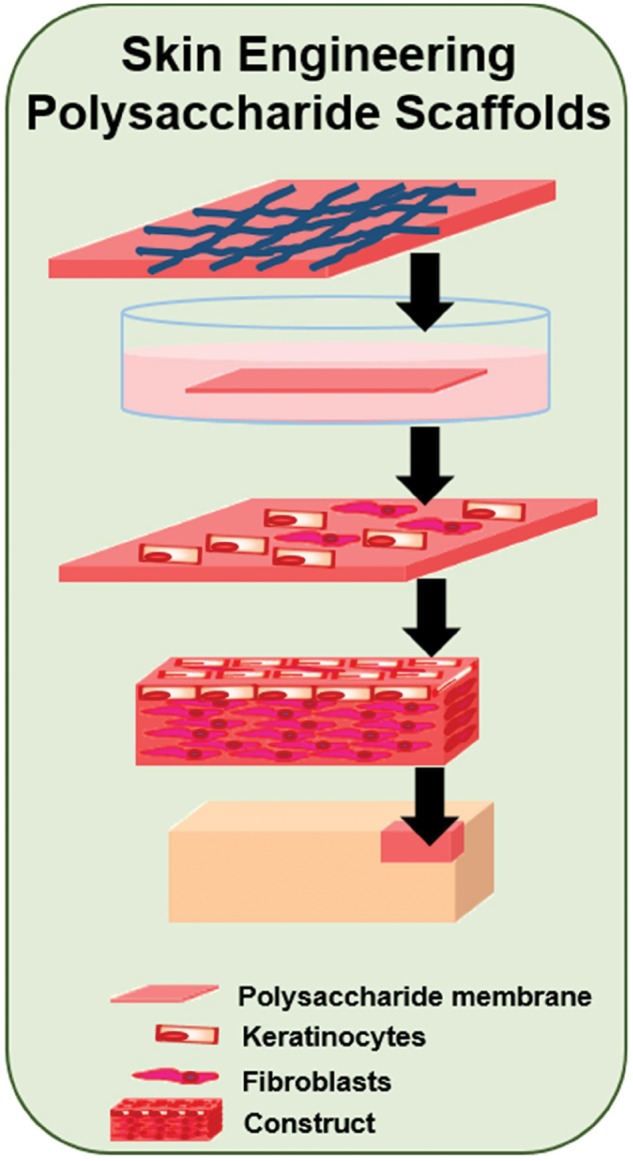
Development of polysaccharide-based constructs for skin wound healing. In the design of cellular constructs for skin healing, polysaccharides have often been used. Skin sheets, membranes or gels have been developed and shown to improve the regeneration rate of the damaged tissue

Polysaccharide-based materials have been used for skin regeneration and wound healing applications as shown in Fig. 6. Azad *et al.* demonstrated that chitosan membranes used as wound dressings enhanced re-epithelialization when compared with the standard of care [95]. Other studies used membranes composed of both chitosan and alginate as wound dressings; and chitosan-based hydrogels as joint wound dressings and drug delivery systems with promising results [96, 97]. Several products containing carbohydrates are already on the market for skin replacement. Cultured skin substitutes commonly utilize hyaluronic acid as part of the scaffold on which keratinocytes and fibroblasts are grown before the graft is placed [93]. While these skin substitutes have seen some commercial success, continual improvements are necessary to decrease the amount of time required before grafting, improve vascularization and mechanical integrity, and scale-up production.

### Cartilage engineering applications

Natural articular cartilage repair is impaired by the avascular nature of articular cartilage, which is composed of chondrocytes and the extracellular matrix produced by these cells. The primary components of the cartilage ECM are type II collagen and GAGs, which include hyaluronic acid, chondroitin sulfate, dermatan sulfate, keratan sulfate and heparan sulfate [98]. The phenotype of chondrocytes determines the composition of the ECM that they produce [26, 98]. Maintaining expression of the appropriate ECM components is essential in properly producing articular cartilage. (An in-depth review of issues in cartilage repair may be found in [98].)

**Figure 7:**
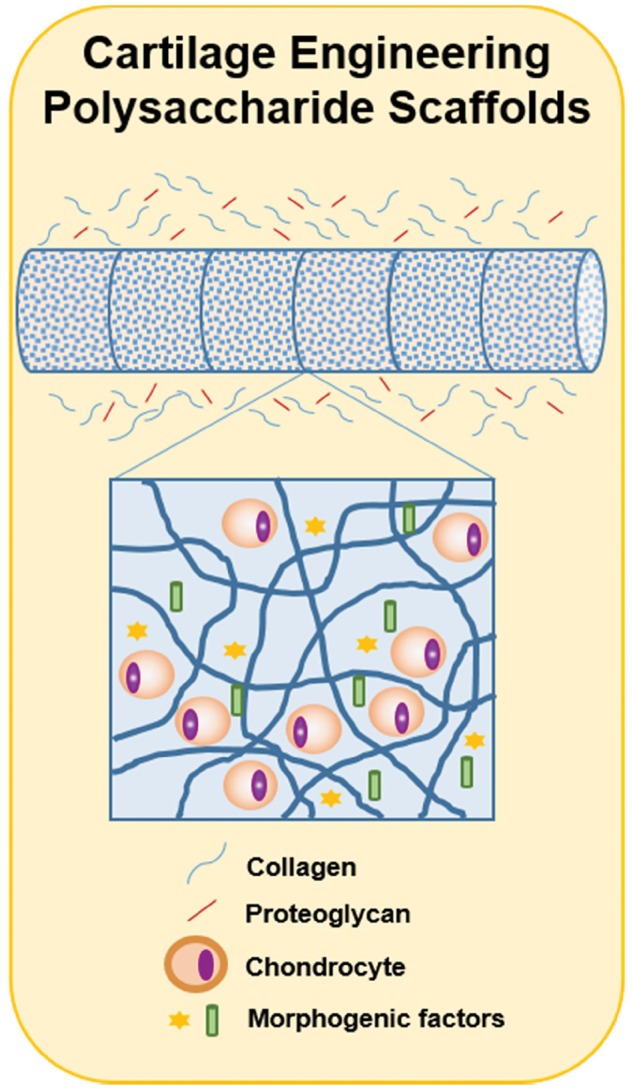
Engineering polysaccharide scaffolds for cartilage repair. The design of scaffolds for cartilage repair has been based on the use of tubular matrices that include morphogenic factors and encapsulation of chondrocytes. These scaffolds secrete collagen and proteoglycans that mimic the ECM of cartilage

Tissue engineering schemes have proposed using bioactive scaffolds to support chondrocyte growth and control the phenotype of chondrocytes (Fig. 7) [99]. Scaffolds may be designed to support ingrowth of chondrocytes from surrounding tissues or be used to encapsulate implanted chondrocytes. The material for these scaffolds must be carefully selected because the chemical and mechanical properties of the scaffold can influence chondrocyte expression and thus the cartilage that is produced [26, 98, 100]. With GAGs being a major component of the chondrocyte extracellular matrix, GAGs and GAG-like materials are commonly mimicked in chondrocyte scaffold design [26]. Several polysaccharide-based biomaterials have been investigated for chondrocyte scaffolding including chitosan, hyaluronic acid and alginates.

Chitosan scaffolds have been the focus of multiple studies because of their biodegradability and GAG-like structure that may mimic the cartilage ECM. Lu *et al.* used *in vivo* studies of chitosan implants to assess its use in cartilage therapeutics. In this work, chitosan was injected into the articular cavity of the knees of Wistar rats [101]. Subsequent results were promising, showing that chitosan increased the number of chondrocytes in the articular cavity and prevented the thickness of epiphyseal cartilage from decreasing compared with the controls [101]. This study demonstrated that chitosan was biocompatible, causing no inflammation, and may contribute chondrogenesis and cartilage repair [101]. Sechriest *et al.* also investigated the application of chitosan in cartilage repair. They showed that chondrocytes cultured on chitosan and chondroitin sulfate-A (a chondroitin sulfate precursor) membranes predominantly produced type II collagen maintained the chondrocyte phenotype [102]. By creating new materials with chondroitin sulfate-A and chitosan, Sechriest *et al.* were able to influence chondrocyte expression toward producing the appropriate type of collagen for articular cartilage ECM. More recently, Masuko *et al.* modified chitosan with a peptide sequence containing RGDS, an adhesion-promoting peptide sequence [103]. Their work demonstrated that conjugated chitosan-RGDSGGC materials increased chondrocyte and fibroblast adhesion and proliferation [103]. Masuko *et al.* also proposed that the procedure used to conjugate RGDSGGC to chitosan could be used to conjugate other peptides and proteins to chitosan [103]. This research showed that tailoring a polysaccharide scaffold with peptides adds more control over cellular adhesion. These studies suggest that further modifications of polysaccharide materials with other molecules, such as proteins, may provide alternate routes toward controlling chondrocyte behavior.

Hyaluronic acid has also been investigated for chondrocyte scaffolding. Unlike chitosan, hyaluronic acid is a natural component of the chondrocyte extracellular matrix, so it has been suggested that hyaluronic acid is an ideal candidate material for chondrocyte scaffolding. It is also biodegradable and biocompatible. Advances have been made in the synthesis of hyaluronic acid-based materials and novel modification methodologies that may expand the potential uses of hyaluronic acid in cartilage engineering. Bulpitt and Aeschlimann developed several methods to couple proteins to hyaluronic acid using amine and aldehyde functionalization [104]. Functionalized hyaluronic acid was crosslinked using esters and aldehydes implanted in a rat subcutaneous model. Results showed that the crosslinker and functionalization of the hyaluronic acid affected inflammation and cartilage generation. The work of Bulpitt and Aeschlimann was novel in that it allowed functionalization and *in situ* polymerization of hyaluronic acid-based materials. More recently, Burdick *et al.* developed a photopolymerizable hyaluronic acid that supported chondrocyte growth and neocartilage formation [29]. Burdick *et al.* improved upon Bulpitt and Aeschlimann’s work by simplifying the synthesis process by using photopolymerization, which can also be carried out *in situ.* It is important to note that other studies have shown that hyaluronic acid-containing biomaterials are biocompatible and contribute to cartilage regeneration *in vivo* [28, 105]. Due to the improvement of its synthesis process and its natural occurrence in cartilage, hyaluronic acid has shown to be an important biomaterial in cartilage tissue engineering.

Several studies have evaluated alginates for use in cartilage scaffolds. Diduch *et al.* encapsulated marrow stromal cells (which produce cartilage like chondrocytes) within alginate, agarose and type I collagen [106]. This study recommended alginate as the optimal material for this application because it had better mechanical properties than agarose and maintained the chondrocyte-like morphology of marrow stromal cells [106]. Caterson *et al.* examined poly(lactic acid) and alginate constructs for marrow stromal cell growth. Results from this study indicated that the poly(lactic acid)/alginate constructs are potential materials for marrow stromal cell encapsulation [107]. Poly(lactic acid)/alginate materials supported chondrogenesis and limited fibrous capsule formation over poly(lactic acid) samples [107]. Iwasaki *et al.* formed an alginate-chitosan hybrid material and studied chondrocyte adhesion and morphology. This work showed that alginate-chitosan materials increased chondrocyte adhesion (over alginate controls) and did not influence chondrocyte morphology [108]. This study demonstrated that the addition of chitosan to alginate materials improved the material properties for cartilage tissue engineering applications.

### Vascular applications

**Figure 8:**
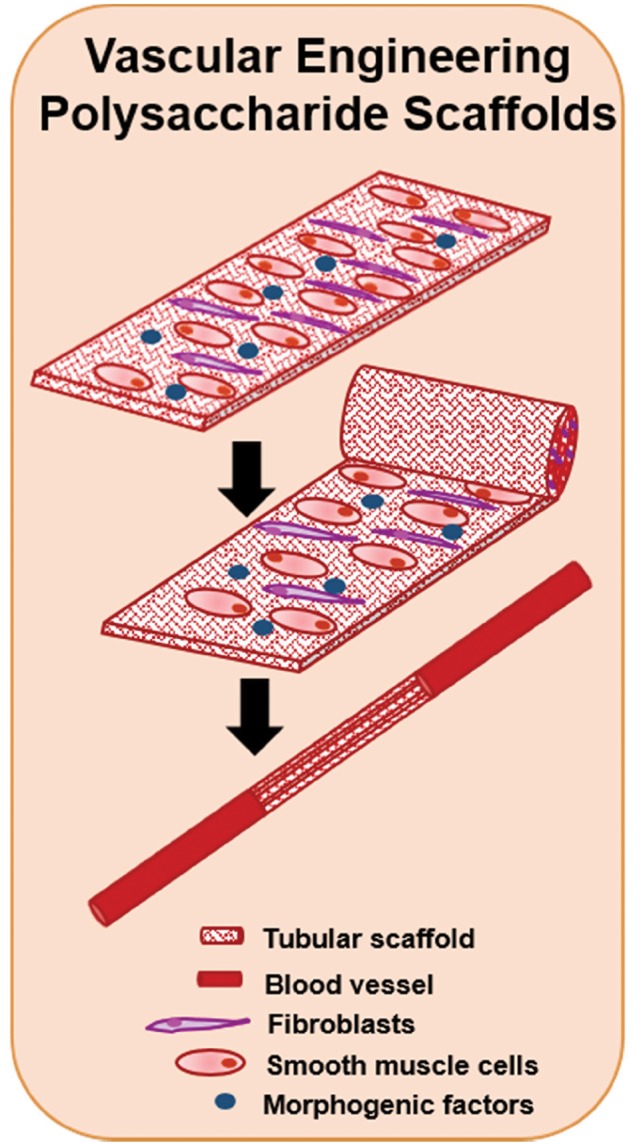
The development of vascular scaffolds using polysaccharides. Tubular scaffolds have been developed for the regeneration of blood vessels using polysaccharide scaffold sheets. A combination of fibroblasts and smooth muscle cells have been seeded with the inclusion of morphogenic factors

Current vascular implants include, but are not limited to, stents and blood vessel grafts. Some of the most important issues in cardiovascular implant engineering include thrombosis, inflammation, re-endothelialization and smooth muscle cell hyperplasia. One of the basic designs of vascular scaffolds is shown in Fig. 8.

Matthew *et al.* investigated chitosan scaffolds for small-diameter blood vessel grafts. Tubular porous chitosan scaffolds were fabricated using several freezing techniques. Pore diameter and the mechanical properties of these scaffolds were controlled by the crystallization temperature [25]. The study showed that tubular scaffolds desirable for cardiovascular implants could be created with a nonporous luminal layer and a porous external layer [25]. Scanning electron micrographs verified that heparin could be incorporated into the chitosan scaffold by rehydrating the scaffold in a heparin solution [25]. A subsequent study expanded on this heparin–chitosan complex. Chitosan scaffolds containing additional GAGs were evaluated for cardiovascular applications. *In vitro* cellular studies showed that the spreading and growth rate of smooth muscle cells and endothelial cells could be controlled by the GAG–chitosan complexes [109]. Smooth muscle cell spreading was more restricted on chitosan and chitosan–GAG structures than the spreading of endothelial cells, which may be additionally beneficial for cardiovascular applications where smooth muscle cell control is of the utmost importance [109]. *In vivo* evaluation of subcutaneously implanted GAG-chitosan scaffolds exhibited tissue ingrowth after 14 days [109]. Of these, heparin-chitosan samples developed a granulation layer containing new vasculature [109]. The ability of chitosan-based biomaterials to elicit different *in vitro* responses of cardiovascular cells, as well as their ability to support tissue ingrowth and angiogenesis *in vivo*, demonstrates that these polysaccharide biomaterials may be further evaluated for use in cardiovascular implants.

### Neural applications

**Figure 9:**
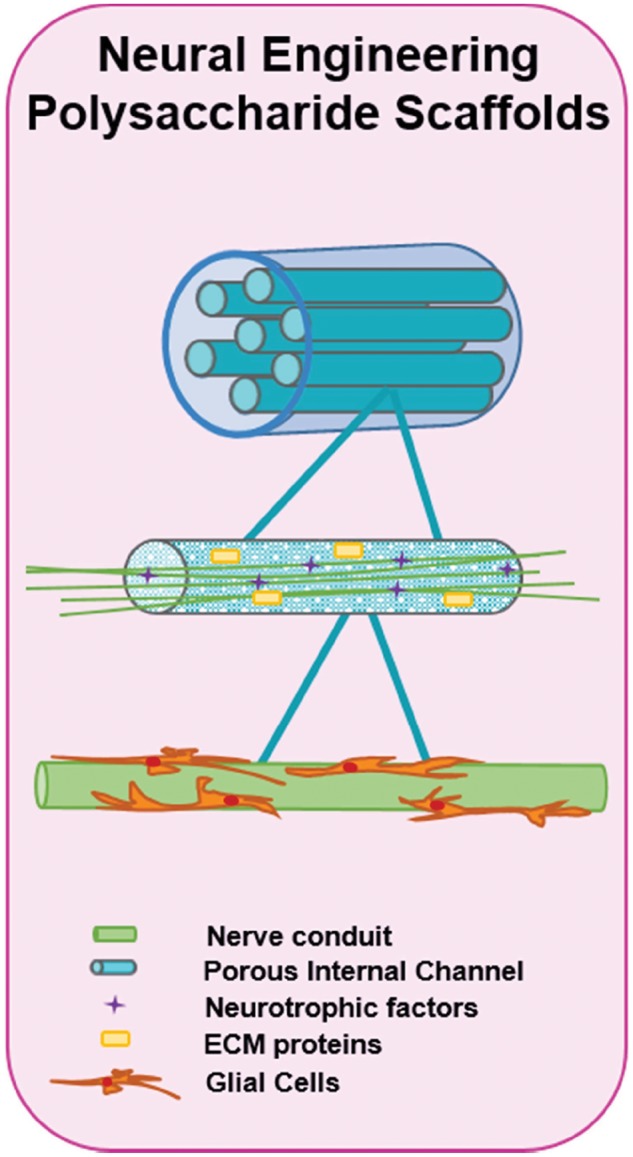
Nerve tissue engineering via polysaccharide scaffolds. The design of neural scaffolds is very complex due to the importance of providing physical guidance for the axons and the prevention of scar-forming tissue. The use of porous guidance channels that include ECM proteins and that release neurotrophic factors is one of the main pieces of neural scaffolds. Within these channels, nerve conduits where glial cells have been seeded, are then inserted

Development of a degradable conduit to facilitate neural regeneration and repair is one of the primary goals in neural tissue engineering (Fig. 9). The material properties of such a channel are essential to the success of the implant. Based on their biocompatibility and mechanical similarities to soft tissue, several polysaccharide-based biomaterials have been investigated for use in such scaffolds for neural reparation.

Cheng *et al.* have investigated chitosan biomaterials for use in nerve guidance channels. This work focused primarily on using chitosan and other moieties, such as gelatin, to create films [110]. The incorporation of gelatin, a protein that can hydrogen bond with chitosan, increased the film’s hydrophilicity and elastic properties [110]. Differentiation of PC12 cells *in vitro* showed that the combined film had an improved affinity for neural cells over chitosan controls [110]. Albumin, collagen and poly-l-lysine were also blended with chitosan [27]. Results indicated that blended chitosan films improved PC12 cell adhesion, differentiation and extension over chitosan controls and non-chitosan blended films [27]. Of these films, the poly-l-lysine-chitosan blend-based film performed the best [27]. It was proposed that increasing the hydrophilicity of the chitosan films with GAGs and poly-l-lysine was responsible for an increased neural cell affinity.

Gu *et al.* studied nerve grafts containing chitosan *in vivo* with promising results [111]. Dual component nerve grafts were fabricated with poly(glycolic acid) (PGA) fibers on the inner surface and an outer layer of chitosan [111]. These conduits were implanted in canines with severed sciatic nerves [111]. After 6 months, the implants had degraded and nerve tissue had formed to reconnect the severed nerve [111]. Dogs that received the chitosan-PGA scaffold recovered similarly to dogs that received an autologous nerve graft [111]. Gu *et al.* proposed that the success of the grafts was due in part to the chitosan component, which allowed blood vessel in-growth, allowed nutrients to enter the graft and served as a barrier to keep out other cells [111]. This study clearly demonstrated that chitosan may be used in neural tissue engineering schemes and illustrates the clinical significance of polysaccharide biomaterials.

Hyaluronic acid as a component of nerve guidance channels has been investigated by Schmidt *et al.* This work took a novel approach to neural biomaterials development by combining natural materials with electrically conductive materials to stimulate neural regeneration chemically, mechanically and electrically [112]. They developed photocrosslinkable hyaluronic acid and polypyrrole films that maintained the electrical conductivity of polypyrrole [112]. The adhesion and morphology of PC12 cells were comparable to controls [112]. These films were also shown to be biocompatible *in vivo* 6 weeks after subcutaneous implantation in a rat model [112]. This study also demonstrated that hyaluronic acid-containing biomaterials have the unique ability to stimulate angiogenesis near the site of the implant [112]. With the capacity to electrically stimulate neurite extension, the ability to mechanically support nerve growth and the capability to promote angiogenesis, these biomaterials are advanced material solutions in neural tissue engineering. Schmidt’s group has clearly showcased hyaluronic acid as a promising biomaterial for neural scaffolding.

### Hepatic applications

**Figure 10:**
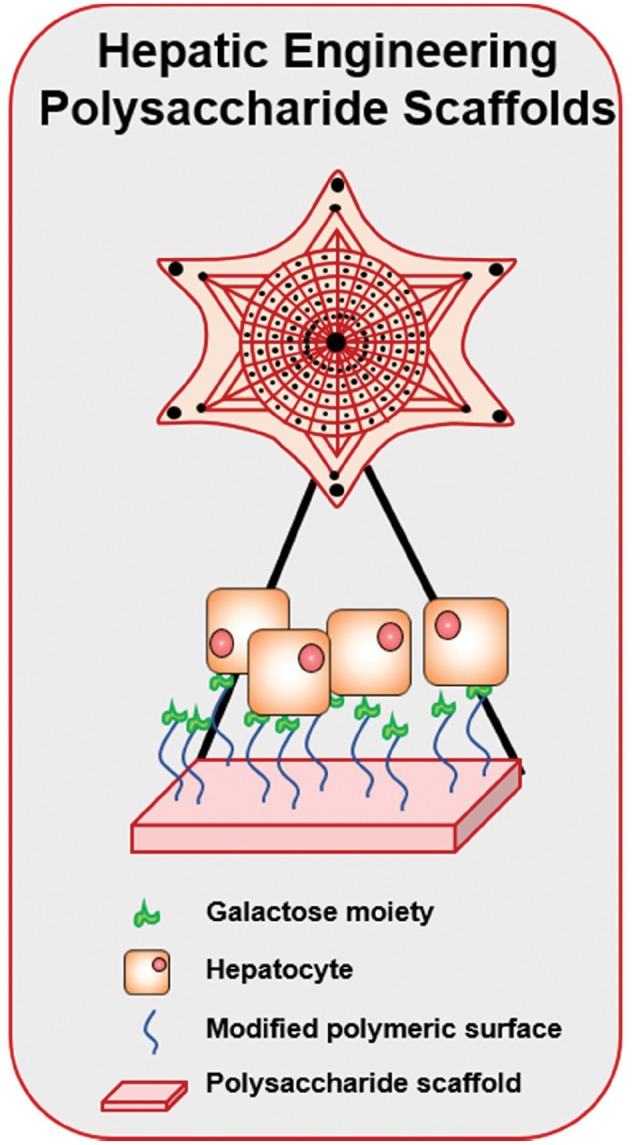
Development of scaffolds for hepatic tissue engineering. The design of adequate scaffolds for hepatic tissue is very difficult due to the high vascularity present in this area. Hence, polysaccharide scaffolds with complex architectures and modified surfaces have been developed. For example, to enhance the differentiation of hepatocytes, the surface of these scaffolds has been decorated with galactose-modified polymers

Hepatic tissue engineering has already begun to incorporate protein-carbohydrate interactions into scaffold design. Hepatic tissue itself is one of the most complex tissues found in the body structurally because of its large amount of vasculature. Control of cell morphology, differentiation and blood vessel ingrowth are essential for hepatic tissue scaffolds. Cho *et al.*, Akaike *et al.* and Griffith *et al.*, have done pioneering hepatic tissue engineering work centered on the fact that liver cells express lectins (asialyglycoprotein receptor (ASGP-R)) with galactose affinities. Therefore, these studies used galactose-containing scaffolds as a natural control mechanism for hepatic tissue engineering as shown in Fig. 10.

Chung *et al.* have examined several galactosylated materials. In one study, they used galactosylated chitosan and alginate scaffolds for hepatic cell growth [113, 114]. They have also examined the effect of different carbohydrate-containing materials on hepatic cell morphology and concluded that the type of carbohydrate used impacted cell spreading [115]. An in-depth review of this work may be found in Ref. [113]. Furthermore, they have shown that the carbohydrate-containing scaffold performs better than alginate controls, suggesting that carbohydrate-containing scaffolds are a potential material for hepatic tissue scaffolds.

Griffith *et al.* focused on the development of synthetic scaffolds modified with galactose. They synthesized poly(ethylene oxide) star polymers modified with galactose for hepatic tissue engineering applications [116, 117]. The results of these studies showed that galactose modification led to specific mediation between the polymer substrate and ASGP-R of hepatic cells [116]. Additionally, galactose-modified polymers improved hepatic cell attachment and growth and proved formidable scaffolds for hepatic tissue engineering [116, 117].

## Advances in polysaccharide biomaterials research: promises for a rich future

Current research is seeking to transform polysaccharide synthesis and screening into higher-throughput processes for the growing field of glycomics. Synthetic polysaccharides are being created as new biomaterials that may act as molecular inhibitors to cellular processes that may be used to create new polymeric biomaterials. This section will outline some of this work and discuss its significance.

### Synthesis

Polysaccharide synthesis has been very complicated in the past. It has relied heavily on complex chemistry because stereospecific and regiospecific control is needed [1]. Protection and deprotection of groups on each carbohydrate are typically performed so that the desired end product is obtained. The enzymatic formation of glycosidic bonds has eased the synthesis process significantly, but efforts to simplify carbohydrate synthesis is still of great need [1]. Emphasis has been placed on the development of solid-phase polysaccharide synthesis procedures, which may improve combinatorial library synthesis by simplifying purification [118–120]. However, high throughput methods that would make polysaccharide chain synthesis as easy as DNA synthesis using PCR would be ideal. Researchers are currently working toward automating this process further [1, 2, 118].

Novel carbohydrate-based platforms have hence been developed to address the need for polysaccharide biomaterials. For instance, Metzke *et al.* devised three innovative saccharide-peptide block copolymers of galactaro–dilysine, galactaro–trilysine and galactaro–tetralysine [121]. The resulting materials demonstrated their biocompatibility and enzymatic degradability in the presence of serine proteases after 5–7 days of exposure. In a different study, Stanek *et al.* have developed a new monomer which contains a carbohydrate: 1,2,5,6-di-*O*-isopropylidene-3-*O*-(*N*-acryloyl-2-methylalaninate)-α-d-glucofuranose (VDMGlu) [122]. This monomer can be crosslinked with methyl methacrylate (MMA), allowing the modulation of its hydrophilicity and allowing copolymers to be formed with MMA, a well-characterized monomer capable of forming hydrogels [122]. In addition to new polymers being created, new molecules for oligosaccharide conjugation are also being synthesized [123].

### Screening: microarrays and libraries

**Figure 11:**
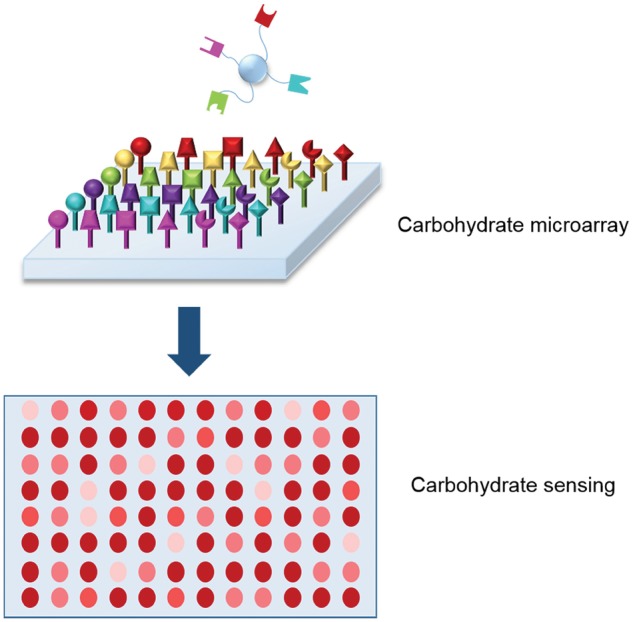
Development of high throughput carbohydrate microarrays. One of the advances in the area of glycomics is the ability to synthesize and evaluate carbohydrate libraries that allow screening and detection of several analytes

Efficient methods for screening polysaccharides are also in development (Fig. 11). Researchers have proposed that carbohydrate microarrays may be used to screen for molecular interactions of interest [2, 124]. Hsu *et al.* have also proposed that lectin microarrays be developed in a similar fashion to probe bacterial interactions [125]. This method allowed rapid and parallel evaluation of changes on bacterial-derived glycans.

Polysaccharide libraries for pharmaceutical screening can already be synthesized [126–128]. Improving their synthesis to yield more diverse libraries with more efficient methods for selecting polysaccharides of interest would greatly enhance glycomics and be of tremendous value to the pharmaceutical industry. Polysaccharide libraries have proven useful tools for selecting carbohydrates. For example, Sofia *et al.* synthesized a disaccharide library from which they were able to extract several disaccharides that inhibit bacterial wall synthesis and show antibacterial potential [129]. Polysaccharide anti-bacterials are promising for the treatment of antibiotic-resistant bacterial infections because they can be tailored to form multivalent bonds that are still likely to be effective even if slight mutations (or evolutions) have occurred.

### Therapeutic development

Current research is aiming to develop polysaccharides that can be used in therapeutic applications. Specifically, they are being investigated to play a role in prodrug therapeutics, which are administered in an inactive form but become activated *in vivo.* These inactive prodrugs are typically formulated as the active drug with a reversibly conjugated molecule which can be removed to activate the drug [130]. Robinson *et al.* have looked at an antitumor prodrug for ‘lectin-directed enzyme-activated prodrug therapy’. Their doxorubicin prodrug relies on the removal of a polysaccharide cap to activate the doxorubicin [131]. Ohya *et al.* have also investigated polysaccharide modification (using dextrose and galactose) as a means to activate an anticancer drug (cisplatin) to target hepatoma cells [132]. Their results suggest that the galactose addition improves hepatoma cell targeting, but subsequent studies on prodrug activation have not yet been published. In a different approach, Wong and Toth outline the use of liposaccharides to create produgs through conjugation [130]. The development of microarrays may help quicken the process of polysaccharide-modified prodrug synthesis toward developing the next generation of prodrugs.

Carbohydrates are being developed to inhibit cancer and viral molecules. For example, Balzarini *et al.* published their work describing findings that lectins from *Cymbidium hybrid*, *Epipactis helleborine* and *Listera ovata* (orchids) can inhibit HIV-1 and HIV-2 [133]. In a different study, Bertozzi *et al.* synthesized a C-linked galactosphingolipid that inhibits the interaction between HIV-1 gp120 and galactosylceramide, a sphingolipid [5]. With the development of combinatorial libraries and higher-throughput systems, selection and synthesis of novel polysaccharides for viral inhibition will become more efficient. These advances, in particular, have a direct impact in the development of targeted delivery of vaccines, as described previously in this work.

Drug delivery systems with better targeting mechanisms will greatly improve the efficacy of therapeutics by increasing bioavailability of a drug locally rather than systemically. Advances in carbohydrate technology may accelerate the development of more specific targeting moieties that will enhance the efficacy of current treatments. Furthermore, biodegradable polysaccharide-based materials are suitable for the treatment of long-term or chronic patients.

**Figure 12:**
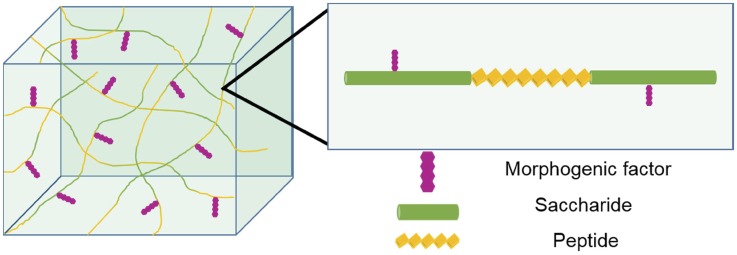
Synthesis of hybrid polysaccharide materials. Improvement of glycomics research has allowed the creation of hybrid materials, where the combination of polysaccharides and other molecules (i.e. peptides) has been achieved. These materials incorporate beneficial characteristics from both components and enhance the versatility of these systems. This approach is very useful, particularly in the development of novel scaffolds for tissue engineering

Synthesis of novel polysaccharide polymers may be designed for particular applications. Hybrid saccharide-peptide copolymers that Metzke *et al.* have devised may be tailored to more closely mimic the extracellular matrix and may serve as new scaffolds for tissue engineering [121]. These copolymers could also be used in drug delivery systems to mimic glycoproteins for the mediation of cell–biomaterial interactions as shown in Fig. 12.

Polysaccharide sequences that can competitively inhibit bacteria via lectin interactions may have a significant impact on antibacterial therapeutics, yet they can have a profound influence on biomaterials design. The use of antibacterial polysaccharides in tissue engineering implants (which are highly susceptible to infection) could limit biofilm formation and subsequent postoperative or post-implant infection.

Another approach currently being explored is the treatment of viruses that have developed resistance to various pharmaceutics with polysaccharides. Infection-inhibiting polysaccharides such as those synthesized by Balzarini *et al.* and Bertozzi *et al.* could become the next anti-viral medications and vaccines [1, 5, 133]. Additionally, polysaccharides can be engineered to form multivalent interactions with viruses that are less affected by small mutations than conventional therapeutics.

Polysaccharides that are being developed to prevent the targeted drug delivery of anti-inflammatory drugs, such as those studied by Eniola *et al.*, have applications in cardiovascular implants [69]. Thrombosis and inflammation are significant difficulties in cardiovascular implants, especially in small diameter grafts as shown by Fukunishi *et al.* [134]. It is hypothesized that the polysaccharides used to mimic neutrophil and leukocyte rolling could also be used to competitively inhibit their adhesion to a biomaterial implant.

Overall, the technology to create a new generation of synthetic polysaccharide materials is in the near future. Chitosan, hyaluronic acid, and alginate have dominated polysaccharide biomaterials as evidenced in the preceding overview of polysaccharide biomaterials. However, the successes of these natural polysaccharides raise the expectations for new synthetic polysaccharides.

## Conclusions and future directions

Polysaccharides have been used in drug delivery and tissue engineering as platforms, targeting moieties and for the selection of active agents. Chitosan-based drug delivery systems have been used for oral gene delivery, hyaluronic acid has been used for anti-tumor therapeutics, while alginates have been predominantly studied for protein drug delivery. Polysaccharide materials have been used as components in tissue engineering scaffolds for cartilage, cardiovascular, neural and hepatic repair.

The burgeoning field of glycomics is developing new higher-throughput methods for synthesizing and studying synthetic and natural polysaccharides. These methods have already led to some fascinating developments regarding antibacterials, antivirals, anti-inflammatory drugs, cancer therapy, cellular adhesion and drug delivery. This is why glycomics is positioned to make a huge impact on biomaterials science. New synthetic polysaccharides and hybrid materials are already beginning to be created. These hybrids will further the development of biomaterials that recognize biological cues with tremendous potential for biomedical sciences. Furthermore, the synthesis of polysaccharides with high modulus and improved mechanical properties will be achieved, with incredible implications for the creation of devices and implants. With the advent of these new polysaccharides, glycomics will make over biomaterials as we know it.

## Funding

This work was supported in part by the National Institutes of Health Grant R01-EB022025, the Cockrell Family Regents Chair, the UT-Portugal CoLab program and Fundação para a Ciência e a Tecnologia, and by the Institute Funds from the Dean of the Cockrell School of Engineering.

*Conflict of interest statement.* None declared.
